# Drosophotoxicology: Elucidating Kinetic and Dynamic Pathways of Methylmercury Toxicity in a Drosophila Model

**DOI:** 10.3389/fgene.2019.00666

**Published:** 2019-08-09

**Authors:** Matthew D. Rand, Daria Vorojeikina, Ashley Peppriell, Jakob Gunderson, Lisa M. Prince

**Affiliations:** ^1^Department of Environmental Medicine, University of Rochester School of Medicine and Dentistry, Rochester, NY, United States; ^2^School of Human Health Sciences, Purdue University, West Lafayette, IN, United States

**Keywords:** Drosophila, methylmercury, Drosophotoxicology, eclosion, toxicokinetics, toxicodynamics, glutamyl cysteine ligase, glutathione

## Abstract

The risks of methylmercury (MeHg) toxicity are greatest during early life where it has long been appreciated that the developing nervous system is an especially sensitive target. Yet, understanding the discrete mechanisms of MeHg toxicity have been obscured by the wide variation in the nature and severity of developmental outcomes that are typically seen across individuals in MeHg exposed populations. Some insight has come from studies aimed at identifying a role for genetic background as a modifier of MeHg toxicity, which have predominantly focused on factors influencing MeHg toxicokinetics, notably, polymorphisms in genes related to glutathione (GSH) metabolism. For example, variants in genes encoding the catalytic and modifier subunits of glutamyl-cysteine ligase (GCLc and GCLm), the rate limiting enzyme for GSH synthesis, have been reported to associate with Hg body burden (Hg levels in blood or hair) in humans. However, GSH can facilitate both toxicokinetics and toxicodynamics of MeHg by forming MeHg-GSH conjugates, which are readily transported and excreted, and by acting indirectly as an anti-oxidant. In this study, we refine a model to distinguish kinetic and dynamic traits of MeHg toxicity using a paradigm of Drosophotoxicolgy. First, we identify that the pupal stage is selectively sensitive to MeHg toxicity. Using a protocol of larval feeding, measurements of Hg body burden, and assays of development to adulthood (pupal eclosion), we identify strain-dependent variation in MeHg elimination as a potential kinetic determinant of differential tolerance to MeHg. We also find that global upregulation of GSH levels, with GCLc trans-gene expression, can induce MeHg tolerance and reduce Hg body burden. However, we demonstrate that MeHg tolerance can also be achieved independently of reducing Hg body burden, in both wild-derived strains and with targeted expression of GCLc in developing neuronal and muscle tissue, pointing to a robust toxicodynamic mechanism. Our findings have important implications for understanding variation in MeHg toxic potential on an individual basis and for informing the process of relating a measurement of Hg body burden to the potential for adverse developmental outcome.

## Introduction

Methylmercury (MeHg) is an environmental toxicant and contaminant of seafood that arises from both natural and anthropogenic sources. The risks of MeHg exposure are greatest during early life, and it has long been understood that the developing nervous system is an especially sensitive target. However, a number of studies have demonstrated a wide variation in neurological outcomes associated with prenatal and early life MeHg exposure, ranging from none at all to measurable motor and cognitive deficits that can be persistent in children through adolescence (Grandjean et al., 1997; Murata et al., 2004; Davidson et al., 2006; Davidson et al., 2008). This wide variation of outcomes seen in humans has hampered the process of risk assessment for MeHg and has called for greater understanding of MeHg toxicity mechanisms. How MeHg manifests toxicity in a developing organism encompasses an extremely complex series of events. Sorting out mechanisms of MeHg toxicity is impingent on characterizing the dose–response relationship, which in turn relies on toxicokinetic principles of absorption, distribution, metabolism, and excretion (ADME). Yet, variability in toxicity can also stem from toxicodynamic mechanisms at the site of action, e.g., affinity for tissue-specific targets and secondary response, such as generation of reactive oxygen species (ROS), both having the potential to vary with genetic background and developmental timing.

The underlying role of genetic background as a modifier of MeHg toxicity has recently received great attention. Studies based in human populations have frequently focused on polymorphisms in genes related to the metabolism of glutathione (GSH) (Llop et al., 2015). GSH can act directly as a conjugate with MeHg to mediate transport, distribution, and excretion (Ballatori and Clarkson, 1982; Ballatori and Clarkson, 1983). GSH also acts as a first line of defense to oxidative stressors and can moderate toxicity stemming from a MeHg insult dynamically by buffering ROS (Shanker et al., 2005; Kaur et al., 2006). These mechanisms predict that polymorphisms affecting GSH synthesis, conjugation, and/or redox status could manifest differences in both MeHg kinetics and dynamics. Supporting evidence for this comes from studies of fish-eating populations harboring polymorphic variants in genes encoding the catalytic and modifier subunits of the glutamyl-cysteine ligase enzyme (GCLc and GCLm, respectively). In several instances, polymorphisms predicting reduced function of GCLc/GCLm show an association with elevated levels of Hg in blood or hair biomarkers (Custodio et al., 2004; Barcelos et al., 2013), consistent with the notion that reduced GSH levels result in slower excretion kinetics and elevated MeHg body burden. Nonetheless, findings across several studies investigating GCLc/m, and other GSH-related genes, including glutathione S-transferases (GSTs) and GSH-dependent ABCC transporters, have produced conflicting results, whereby associations with both higher and lower Hg levels in blood or hair are seen for the same polymorphic variant (Custodio et al., 2004; Schlawicke Engstrom et al., 2008; Barcelos et al., 2013). Furthermore, associations of GSH-related gene polymorphisms with neurodevelopmental outcomes are also reported that are independent of association with Hg levels in biomarkers (Engstrom et al., 2016; Wahlberg et al., 2018), pointing to roles for toxicodynamic mediators of MeHg toxicity. These variable findings have highlighted the need to discern fundamental toxicokinetic versus toxicodynamic mediators of MeHg toxicity.

**Figure 1:**
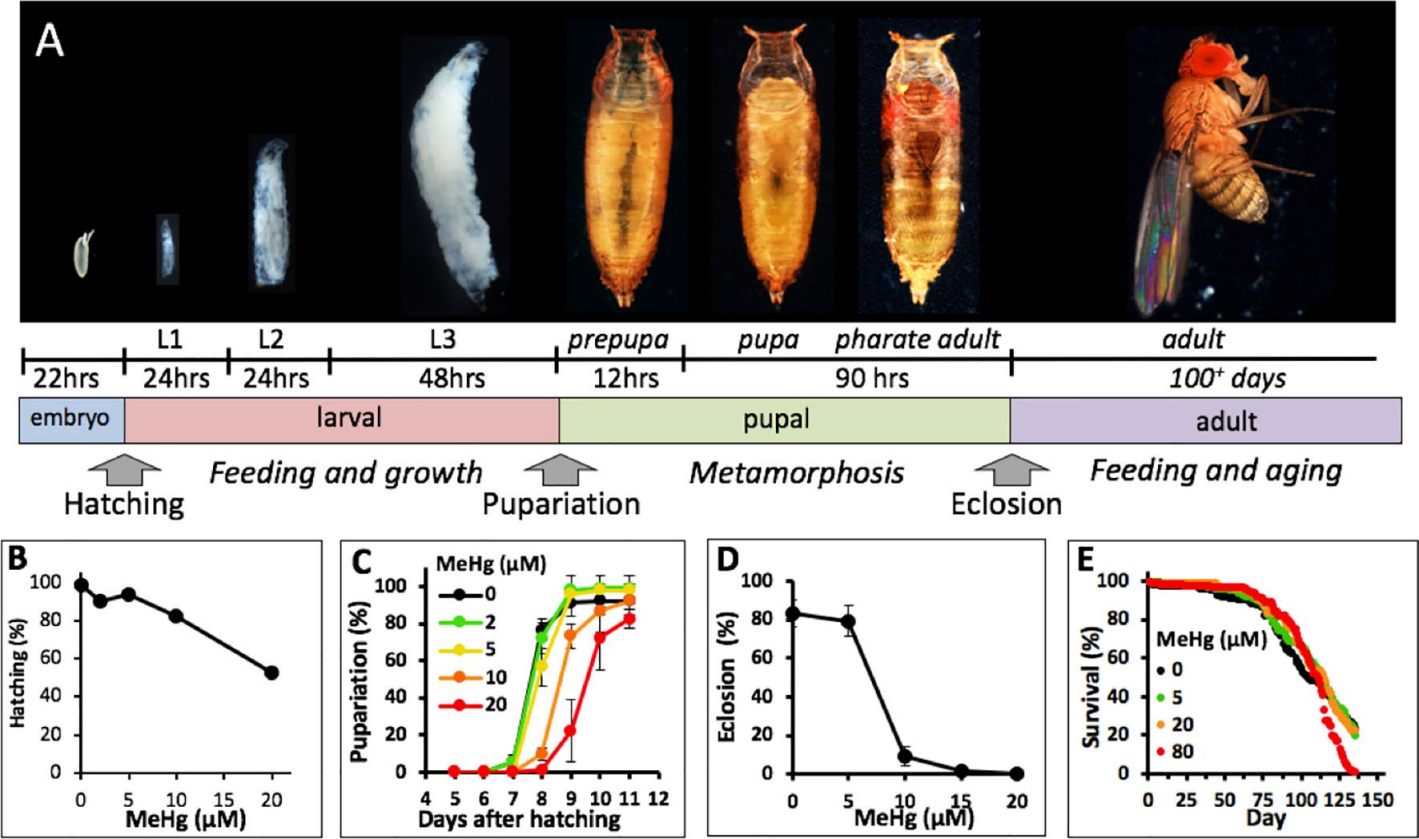
Drosophila life cycle and toxicity endpoints of MeHg. **(A)** Drosophila development from embryo to adult is holometabolous and progresses through intermediate larval and pupal stages. Approximate development times for each stage at 25°C and transitional events that can be scored (e.g., hatching, pupariation and eclosion) are indicated. **(B–D)** Various endpoint assays for the effects of developmental MeHg exposure. Canton S flies were used in all assays. **(B)** Larval hatching rate of embryos collected from a mating population of flies fed on the indicated concentration of MeHg food (Reproduced from Rand et al., 2008). **(C)** Rate of pupariation of larvae reared from the L1 stage on the indicated concentration of MeHg food. **(D)** Rate of eclosion of pupae reared on MeHg food at the indicated concentration throughout the larval stages. **(E)** Survival rate of adult flies reared on MeHg-free food from the larval stage and transferred to food with indicated MeHg concentration after 2–3 days after eclosion.

A number of recent studies have turned to the Drosophila model to elaborate MeHg toxicity mechanisms. Drosophila have several powerful attributes for toxicological studies including a high degree of genetic conservation of fundamental signaling and structural protein networks and an extensive array of molecular genetic tools for both forward and reverse genetic approaches (reviewed in Rand, 2009; Chifiriuc et al., 2016). From a developmental toxicology perspective, its holometabolous life cycle offers a unique opportunity to assay toxicant efficacy at four distinct life stages, embryo, larva, pupa, and adult, and subsequently monitor a variety of endpoints, such as egg laying, embryo hatching, larval growth and locomotion, pupa formation and eclosion, adult lethality, longevity, as well as several complex behaviors (**Figure 1A**).

Studies of MeHg with flies have illuminated several new perspectives on MeHg toxicity. Importantly, experiments by Magnusson and Ramel were the earliest investigations to explain variation in MeHg toxicity by a genetic component (Ramel and Magnusson, 1969; Magnusson and Ramel, 1986). Remarkably, this early work has consolidated the hypothesis that specific genetically controlled mechanisms for moderating MeHg toxicity stand apart from toxicity of other metals, such as lead and cadmium (Magnusson and Ramel, 1986). Using a paradigm of larval feeding and assays of successful development to adulthood (pupal eclosion), a dominant polygenic trait of tolerance to MeHg was identified in wild and in laboratory-selected strains of flies (Magnusson and Ramel, 1986), an observation that has since been reinforced by Mahapatra et al. (2010). Magnusson and Ramel (1986)also evaluated MeHg uptake and excretion and found that susceptible strains accumulated MeHg to higher levels, implicating a toxicokinetic basis for the variability in tolerance.

Recent strategies using Drosophila have attempted to characterize complex behavioral traits that are susceptible to developmental MeHg exposures, such as courtship and mating behavior (Chauhan et al., 2017), locomotor activity, and circadian rhythm (Algarve et al., 2018), with the notion that these reflect neurotoxic effects. Additional insight has come from investigations of combinatorial exposures with other toxicants and antioxidants, such as alcohol and selenium (Chauhan and Chauhan, 2016; Leao et al., 2018). Many of these MeHg-induced deficits have shown to track with production of ROS and corresponding oxidative stress markers, contributing to a rationale for preventative strategies *via* endogenous or exogenous antioxidant enhancers. Nonetheless, these findings have produced little advance in understanding of MeHg-specific pathways, as ROS production is an endpoint common to numerous toxicants.

Mechanistic insight into MeHg toxicity has come from two additional strategies using Drosophila: a candidate gene approach to interrogate effects of known or suspected genes or pathways and an unbiased screening approach to identify gene candidates *via* transcriptomics or genomic methods. Candidate genes have been examined using the GAL4-UAS transgene expression system (Brand et al., 1994) to target overexpression or knockdown genes of interest in tissue-specific and developmental stage-specific patterns. For example, using eclosion assays with transgene expression in flies, we have demonstrated a MeHg moderating activity for conserved members of Phase I (CYPs) (Rand et al., 2012), Phase II (GSTs) (Vorojeikina et al., 2017), and Phase III (MRP/ABCC1) (Prince et al., 2014) xenobiotic metabolism genes. Through transcriptomic screens of MeHg-exposed fly embryos and larvae, we have identified candidates within the Notch receptor pathway, Cytochrome p450 family, and the innate immunity pathway that moderate MeHg toxicity (Bland and Rand, 2006; Rand et al., 2009; Mahapatra et al., 2010; Engel et al., 2012; Mahapatra and Rand, 2012; Rand et al., 2012; Engel and Rand, 2014). With a genome-wide association screen we revealed genes in myogenic and muscle development pathways that associate with effects of developmental MeHg exposure on eclosion (Montgomery et al., 2014). Despite resolving strong MeHg-protective effects of individual gene candidates in tissue-specific patterns through these combined efforts, the underlying mechanisms of MeHg toxicity remain enigmatic. For example, Vorojeikina et al. (2017) found that elevated GST activity in the fat body (an organ with liver-equivalent function) or the gut of developing flies can rescue MeHg-inhibited eclosion. Yet, whereas GST overexpression in the fat body causes a significant reduction in Hg body burden, GST expression targeted to the gut shows no change in MeHg body burden relative to control flies (Vorojeikina et al., 2017). This contrasting profile suggests that the specificity with which MeHg acts can be fundamentally sorted to kinetic or dynamic pathways.

Here, we re-examine the paradigm of developmental MeHg toxicity in the Drosophila model with an overall aim of distinguishing genetic differences that track with properties of toxicokinetics and toxicodynamics. Comparative sensitivity to MeHg at distinct stages across the life cycle is evaluated. Kinetics of MeHg uptake and excretion are characterized to identify determinants of Hg body burden. Strain variation in MeHg body burden and GSH levels are related to naturally occurring and genetically induced MeHg tolerance traits in wild and transgenic flies expressing GCLc, respectively. Our findings point to genetically controlled traits that can moderate MeHg toxicity *via* either kinetic or dynamic pathways that can be differentially expressed in individuals and obscure the relationship of body burden and developmental outcome.

## Methods

### Drosophila Stocks

The following *Drosophila* strains were obtained from the Bloomington Drosophila Stock Center (Indiana University, Bloomington, Indiana): Canton S (CS, #1), w[1118] (#5905); Hikone R (#4267), Mef2GAL4 (#27390, pan-muscle driver); ELAVGAL4 (#8760, pan-neural driver); UASGFP-CD8 (#5130, plasma membrane localized GFP). The DGRP “Raleigh” lines are all available at the Bloomington Stock Center. NP1GAL4 (gut epithelial driver) and ActinGal4/Cyo (ubiquitous driver) were a gift from Benoit Biteau, Univ. of Rochester, and the UASGCLc (line #6, glutamyl-cysteine ligase catalytic subunit) was a gift from William Orr, Southern Methodist Univ., Texas. Flies were kept on a 12/12-h light/dark cycle in a 25°C humidified chamber on a standard fly food made of cornmeal, molasses, yeast, and agar.

### MeHg Developmental Toxicity Assays

#### Embryo Hatchings

Effects of embryonic MeHg exposures were scored by the rate of larval hatching as reported previously (Rand et al., 2008). Briefly embryos from a mating population of Canton S flies reared on either control or MeHg containing food (0–20 µM) were collected, transferred to fresh grape-agar medium plates, and developed for 24 h. Hatching of first instar larvae was determined manually under a stereo dissecting microscope 24 h after transfer of embryos to the new plate. Between 260 and 643 embryos were scored for each concentration of MeHg.

#### Larval Pupariation

Pupariation (formation of the pupa) rates were determined in 50-ml culture vials containing 10 ml of control or MeHg food. Briefly, first instar Canton S larvae were collected from embryos obtained within a 2–4 h laying period from a mating population of 150 to 300 flies and aged to L1 stage. L1 Canton S larvae were transferred to vials (n = 50/vial) and allowed to develop at 25°C. Newly formed pupae were counted daily until day 11 after larval hatching. All determinations were done with a minimum of triplicate vials and reported as percent pupariation expressed as the mean and standard deviation.

#### Pupal Eclosion

Eclosion (emergence of adults from the pupa case) rates were determined as previously described (Rand et al., 2014). Briefly, L1 Larvae were seeded at 50/vial on food (Jazz Mix, Fisher Scientific, #AS153) containing 0 to 20 µM MeHg (methylmercury chloride, Sigma-Aldrich # 215465), and allowed to develop for 13 days. Flies that successfully eclosed were scored and expressed as percent eclosion. Eclosion rates for DGRP flies are expressed as an eclosion index, as previously reported (Montgomery et al., 2014). Briefly, the eclosion index is simply a summation of the normalized percent values for eclosion at each of the three MeHg concentrations: 5, 10, and 15 µM.

#### Adult Longevity

MeHg effect on adult fly lifespan was determined by rearing newly eclosed male Canton S flies (2–3 days after eclosion) on food containing various concentrations of MeHg (0–80 µM). Flies were reared in five replicate vials with between 20 and 45 flies per vial and a total of 150 flies per MeHg concentration tested. Flies were transferred to fresh food every 3 to 4 days at which time dead flies were counted. Survival curves were analyzed by log rank tests (Mantel-Cox) using Prism (Graphpad, San Diego).

### Timing of Developmental MeHg Exposure

Canton S and Hikone R strains were tested in parallel to examine effects across two previously established MeHg susceptible and tolerant strains (Rand et al., 2014). L1 larvae were collected from timed layings of embryos of mating populations of each strain. Food (Jazz Mix) was prepared with various concentrations of MeHg (0–40 µM) and poured in 10-cm plastic dishes for easier manipulations of the larvae. Larvae were manually placed on control or MeHg food medium to accomplish exposures spanning windows of 24 to 72 h falling within the larval developmental stage (∼96 h total). Larvae were cultured in the dishes with the lids on at 25°C. Early exposure (i.e., L1 stage for 24 h) was followed by recovery on medium without MeHg and likewise, late exposures (L2 and L3 stage) were preceded by rearing larvae from the L1 stage on MeHg-free food. Eclosion rate was scored as described above.

### MeHg Absorption and Elimination Kinetics

Canton S and Hikone R L1 larvae were collected from timed layings of embryos of mating populations of each strain and aged to the L2 stage. Larvae (∼500) were placed on 5 µM MeHg food (Jazz Mix) prepared in 10-cm plastic dishes for easier manipulation and incubated at 25°C. At various time points over 48 h larvae were sampled (n = 15–20 per time point), rinsed in phosphate buffer to remove extraneous food, blotted dry to remove buffer, and frozen for 30 min to kill and immobilize the larvae. Larvae were then weighed, and total mercury was determined on pooled samples of 15 to 20 larvae each by thermal decomposition, Hg amalgamation, and atomic absorption using a DMA-80 Direct Mercury Analyzer (Milestone, Shelton CT). Hg content was then expressed in µg/g [parts per million (ppm)] on a wet weight basis. MeHg uptake analyses were performed on three replicates of mating populations of each strain. The average and standard deviation for three trials were plotted.

For elimination rate analyses, early L2 larvae were fed on 5 µM MeHg food for 40 h to reach near steady-state levels of MeHg and transferred to MeHg free food for recovery. At various time points over 48 h larvae were sampled (n = 15–20 per time point), rinsed in phosphate buffer to remove extraneous food, and frozen. Total Hg determinations were done on weighed samples of larvae as described above, and total Hg content was expressed in µg/g (ppm) on a wet weight basis. Elimination analyses were performed on two replicates of mating populations of each strain. Elimination was expressed as decrease in total Hg (tHg) on a ppm basis over time. Due to the rapid growth of larva in the L2 and L3 stage (a 3–15-fold increase in mass in a 48-h window was observed), the decrease in Hg on a ppm basis was largely affected by growth dilution. Therefore, the projected decrease due to growth dilution was calculated and plotted on the same axes. Due to the variance in starting Hg levels and growth rate between trials and strain, data for each trial are presented separately.

### MeHg Body Burden Biotransformation, and Distribution

MeHg body burden resulting from MeHg dosing throughout the larval stage was determined by measuring tHg in one to three replicates of pooled samples of 10–40 larvae or pupae for each replicate. For whole-body analyses larvae were collected at indicated developmental time points and pupae were typically collected between 12 and 24 h after pupa formation (APF). For determinations of Hg in body regions (head, thorax, abdomen) pupae were collected between 72 and 96 h to allow for morphogenesis of internal organs. Body regions from 10 pupae were separated by transection with a clean razor blade and pooled for Hg analysis. Total Hg was determined using a DMA-80 Direct Mercury Analyzer and determined in µg/g on a wet weight basis expressed in ppm.

MeHg biotransformation (demethylation) was determined at both the larval and pupal stages by measuring tHg and iHg in each sample according to the Magos method (Magos and Clarkson, 1972). Biotransformation was interpreted from an increase in proportion of iHg:tHg in the sample relative to the MeHg added to the food source. In rodents, MeHg biotransformation is known to occur in the gut where bacteria are responsible for demethylation (Rowland et al., 1978). Due to the non-sterile nature of larval cultures, with animals immersed in the food and continually feeding and excreting, we predicted that biotransformation by bacteria in the animal or the food would be difficult to distinguish. Therefore, for the larval stage, determinations were done on the whole cultures of food and larvae combined. For this, “dense” cultures of 50 L1 larvae in 500 µl of food (Jazz mix) containing 0, 5, 10, 15 µM MeHg were prepared and incubated for 4 days at 25°C. In addition, food without larvae was cultured at 25°C in parallel. Food preparations were also stored at −20°C for 4 days and analyzed for iHg and tHg in parallel. The weight of the cultures was determined and the entire culture was then homogenized and base hydrolyzed for differential analysis of iHg and tHg by the cold vapor atomic adsorption (CVAA) method (Magos and Clarkson, 1972). Hg values were determined on a ppm weight basis. Biotransformation in the pupal stage was determined with pupae previously reared on 5 or 10 µM MeHg food throughout the larval stages. Pupae were collected at the late stage (80–96 h APF). Pooled samples of 10 pupae were weighed and then homogenized and base hydrolyzed for differential analysis of tHg and iHg by the CVAA method (Magos and Clarkson, 1972). Determination of %iHg (iHg/tHg *100) in the samples were compared to the value of %iHg in the stock solution of MeHg used to prepare the food, which was determined to be 4.97% iHg by the Magos method. It is noted that the Magos method has been shown to give an ∼5% error in iHg determination in samples that are known to be 100% MeHg (Lind et al., 1994).

### Pharmacological and Genetic Rescue of Eclosion

L-methionine has previously been shown to antagonize MeHg uptake in cells and animal models (Aschner and Clarkson, 1989; Caito et al., 2013), which is consistent with a blocking of large amino acid transporters (LAT) known to meditate uptake of MeHg-cysteine conjugates. Effects of L-methionine (Sigma #M9625) on eclosion and on MeHg uptake were tested with Canton S and Hikone R larvae reared on MeHg containing food with or without 1 to 10 mM l-methionine added. Eclosion rates were determined as described above. Total Hg was measured in three replicates of pooled samples of 10 pupae collected 12 to 24 h APF. Total Hg was determined using a DMA-80 Direct Mercury Analyzer.

Glutathione (GSH) is known to conjugate with MeHg and attenuates MeHg toxicity by enhancing transport and excretion (Kerper et al., 1996). Genetic rescue of eclosion was therefore evaluated with induced expression of the glutamyl-cysteine ligase catalytic subunit (GCLc), a rate limiting enzyme in the GSH synthesis pathway (Orr et al., 2005). Transgene expression of GCLc was accomplished using the Gal4-UAS system (Brand et al., 1994) with various combinations of crosses of virgin GAL4 females (n = 100–150) with UAS males (n = 50–100) to generate L1 larvae progeny to be assayed for eclosion and Hg body burden. GAL4 drivers include ActinGAL4 (ubiquitous expression), ELAVGAL4 (pan-neural driver), and Mef2GAL4 (pan-muscle driver). Crosses were done with the UASGCLc responder line and the w[1118] as a control strain. GCLc activity was determined indirectly by measuring GSH levels in pupae from the ActinGAL4 > UASGCLc and comparing to levels in the ActinGAL4 > w[1118] control progeny pupae. GSH determinations were done on pupal homogenates (n = 10 pupae/sample) by colorimetric determination of reduced GSH by a two-step chromophoric thione reaction (Biooxytech GSH-400, #21011). GSH levels were expressed as millimolar concentration based on the wet weight of the pupae.

### Oxidative Stress Measurements

The 2,7-dichlorofluoresceindiacetate (DCF-DA) reagent (Sigma) was used as a global indicator of oxidative stress since DCF-DA is known to be responsive to a variety of ROS (Chen et al., 2010). DCF-DA assays were performed with adaptations from previously published protocols (Chauhan et al., 2017; Leao et al., 2018). DCF-DA stock solution was prepared at 2.5 mM in DMSO and used at 2.5 µM final concentration. Pupae (10 per replicate) were homogenized in 200-µl phosphate-buffered saline (PBS) and raised to 1 ml final volume and the lysate centrifuged at 10,000 × g for 10 min at 4°C. A total of 40 µl of lysate was assayed in a final volume of 200 µl of DCF-DA and incubated for 80 min at room temperature in the dark. Fluorescence intensity (485 nm excitation and 530 nm emission) was determined in a Biotek fluorescence plate reader with Gen5 software (Biotek). Total protein in the lysates was determined in parallel using the BCA protein assay reagent (Pierce). Fluorescence intensity was normalized to total protein content in each sample. Three replicates of 10 pupae were assayed for each treatment condition.

### Statistics

For eclosion assays, a two-tailed z-test was conducted, as the percent of flies successfully eclosed is a non-continuous value reaching 0% and 100% at the minima and maxima, respectively. Each MeHg concentration was treated categorically by comparing respective genetically manipulated strains or GAL4 > UAS crosses to their relevant control strain or cross, as indicated. All values are represented as an average of three replicates plus and minus standard deviation. *p* Values less than or equal to 0.05 were considered significant.

For mercury body burden, three replicate Hg determinations were done for each MeHg concentration for each strain (Canton S and Hikone R) and for each GAL4 > UAS cross combination. Pairwise t-test was used to determine significant differences between strains (e.g., Canton S and Hikone R), treatments (e.g., +/- L-methionine), or genetic background (e.g., ActinGAL4 > GCLc versus ActinGAL4 > w[1118]). *p* Values of less than 0.05 or equal to were considered significant.

Correlations between eclosion index, body burden, and GSH levels among the DGRP isogenic wild strains were done with linear regression statistics. Analyses were performed using the Prism software (Graphpad, San Diego). Effects of upregulating GCLc on Hg distribution in each body part were analyzed by the Wilcoxon-rank test because of unequal variances between the samples and non-parametric sample distributions. One-way chi-square approximation test statistic was then used to derive a *p* value with *p* < 0.05 considered significant.

The significance of interaction between genotype (Actin > w1118 and Actin > GCLc) and MeHg concentration with respect to DCF fluorescence was analyzed by a two-way ANOVA using the statistical software JMP Pro 14. Because no interaction was determined, a one-way ANOVA method was used to compare mean random fluorescence intensities between MeHg concentrations for each genotype.

## Results

### Development During the Larval to Adult Transition Is the Most MeHg-Sensitive

A number of studies investigating developmental effects of MeHg in Drosophila, including our own, have utilized the decrease in eclosion rate as a toxicological endpoint (Magnusson and Ramel, 1986; Rand et al., 2014). With the objective of characterizing MeHg-specific pathways we reasoned that it would be best to interrogate the most vulnerable window of MeHg toxicity across the Drosophila lifecycle. Using the standard Canton S laboratory strain, we examined a variety of development endpoints subsequent to exposure to MeHg food at three distinct life stages: embryonic (parental feeding), larval, and adult (**Figure 1A**). We previously reported MeHg effects on Drosophila embryos examining rates of hatching of larvae from embryos of a mating population of flies fed on MeHg food (Rand et al., 2008). The data are presented again here for comparison. Embryos revealed a dose-dependent decrease in hatching ability with a 50% reduction in hatch rate seen with 20 µM MeHg treatment (**Figure 1B**). With MeHg exposure initiated at the first instar larval stage (L1) a dose-dependent delay in time to pupariation was seen with approximately a 1-day delay seen on 10 µM MeHg food and a 2-day delay on 20 µM MeHg food relative to untreated larvae (**Figure 1C**). No significant decrease in the overall pupariation rate was observed at day 11 after embryo hatching. In contrast, approximately 90% of developing flies failed to eclose subsequent to larval feeding on 10 µM MeHg food (**Figure 1D**). Complete inhibition of eclosion was observed with rearing of larvae on 15 µM MeHg and higher concentrations (**Figure 1D**). Flies developed from larvae reared on MeHg-free food and exposed to MeHg as adults showed comparatively less sensitivity to MeHg where the median survival rate was nearly equivalent on all concentrations of MeHg food up to 80 µM with no significant difference between the 0- and 80-µM MeHg exposures (**Figure 1E**). These data indicate that developmental events during the pupal stage that culminate in eclosion are the most susceptible to MeHg toxicity.

### Eclosion Sensitivity Relates to Timing of MeHg Exposure

**Figure 2:**
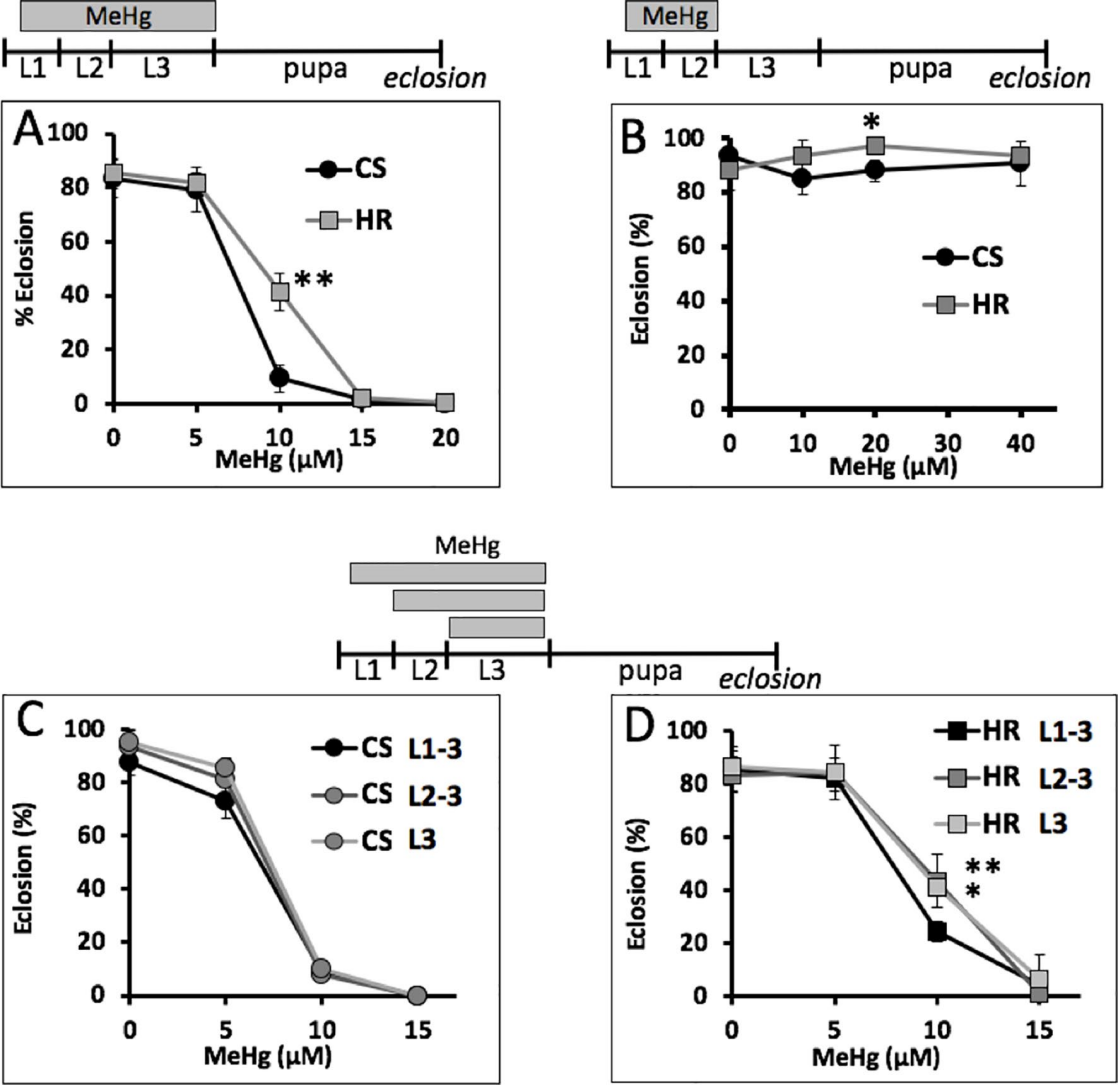
MeHg-sensitive windows of exposure. Eclosion rate for Canton S (CS) and Hikone R (HR) larvae exposed to MeHg at the indicated times spanning all or portions of L1–L3 development are shown. **(A)** Eclosion rate with MeHg exposure over entire larval period. **(B)** MeHg exposure for the L1–L2 stage followed by recovery and development on MeHg-free food. **(C, D)** Comparison of eclosion rates with restricting exposure to L2–L3 stage (*p = <0.05, **p = <0.01, z-test).

To further resolve the most MeHg-sensitive window of development, we exposed larvae to MeHg within discrete developmental periods and subsequently assayed eclosion rate. Two strains, the Canton S and Hikone R, were compared whereby the latter has previously been shown to have higher tolerance to MeHg (**Figure 2** and Rand et al., 2014). Exposure to MeHg during the L1–L3 larval stages results in dose-dependent inhibition of eclosion, with Canton S exhibiting approximately 10% eclosion compared to 40% eclosion seen for Hikone R on 10 µM MeHg food (**Figure 2A**). When MeHg exposure is restricted to the L1–L2 larval stages there was no overall effect on subsequent eclosion rate at exposures up to 40 µM MeHg (**Figure 2B**). In contrast, if MeHg exposure was restricted to the L2–L3, or just the L3 stage, eclosion rates for the Canton S decline in a concentration-dependent manner with the same profile as that seen with MeHg exposure initiated at L1 (**Figure 2C**). A similar profile was seen for the Hikone R strain, except that eclosion rate was slightly less inhibited when MeHg exposure was limited to the L2–L3 stages (**Figure 2D**). These data demonstrate that MeHg sensitivity is related to exposures occurring in the late larval stage.

### Influence of MeHg Absorption and Elimination on Body Burden in the Pupae

**Figure 3:**
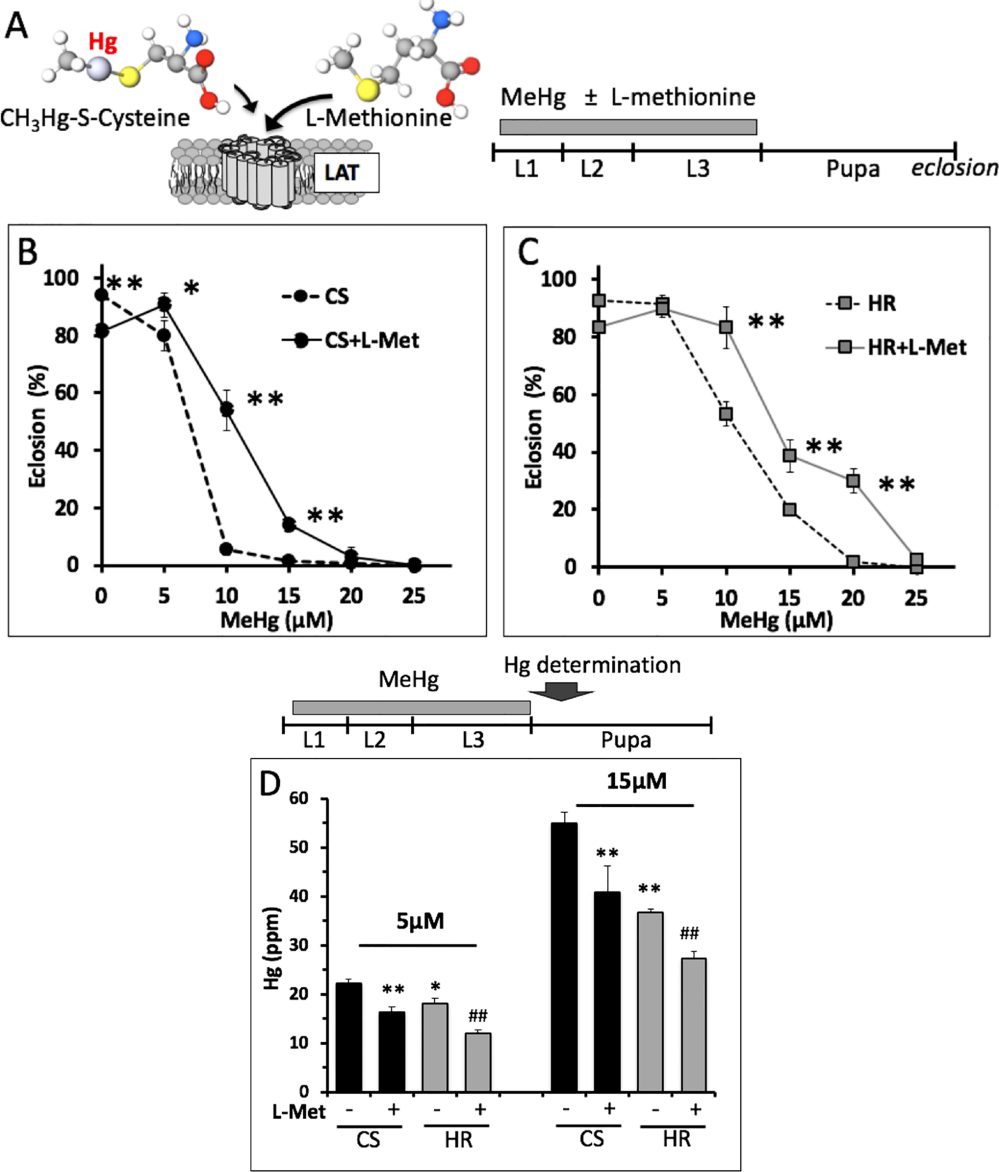
L-methionine effect on eclosion rate and body burden with MeHg exposure. **(A)** L-methionine and MeHg-cysteine conjugate are both substrates for the large amino acid transporter (LAT) (Aschner and Clarkson, 1989). L-methionine can act as a competitive inhibitor of MeHg uptake. **(B, C)** Eclosion rates of Canton S (CS) and Hikone R (HR) strains were determined after exposure to MeHg food with or without 5 mM L-methionine throughout larval stages (*p = <0.01, **p = <0.001, z-test). **(D)** Total Hg was determined in CS and HR early stage pupae (12–24 h APF) after exposure to the indicated concentration of MeHg in the presence or absence of 5 mM L-methionine throughout the larval stages (n = 3 replicates of pooled samples of 10 pupae; t-test; *p = <0.05, **p = <0.005 relative to CS; ^#^ *p* < 0.05, ^##^ *p* < 0.005 relative to HR).

**Figure 4:**
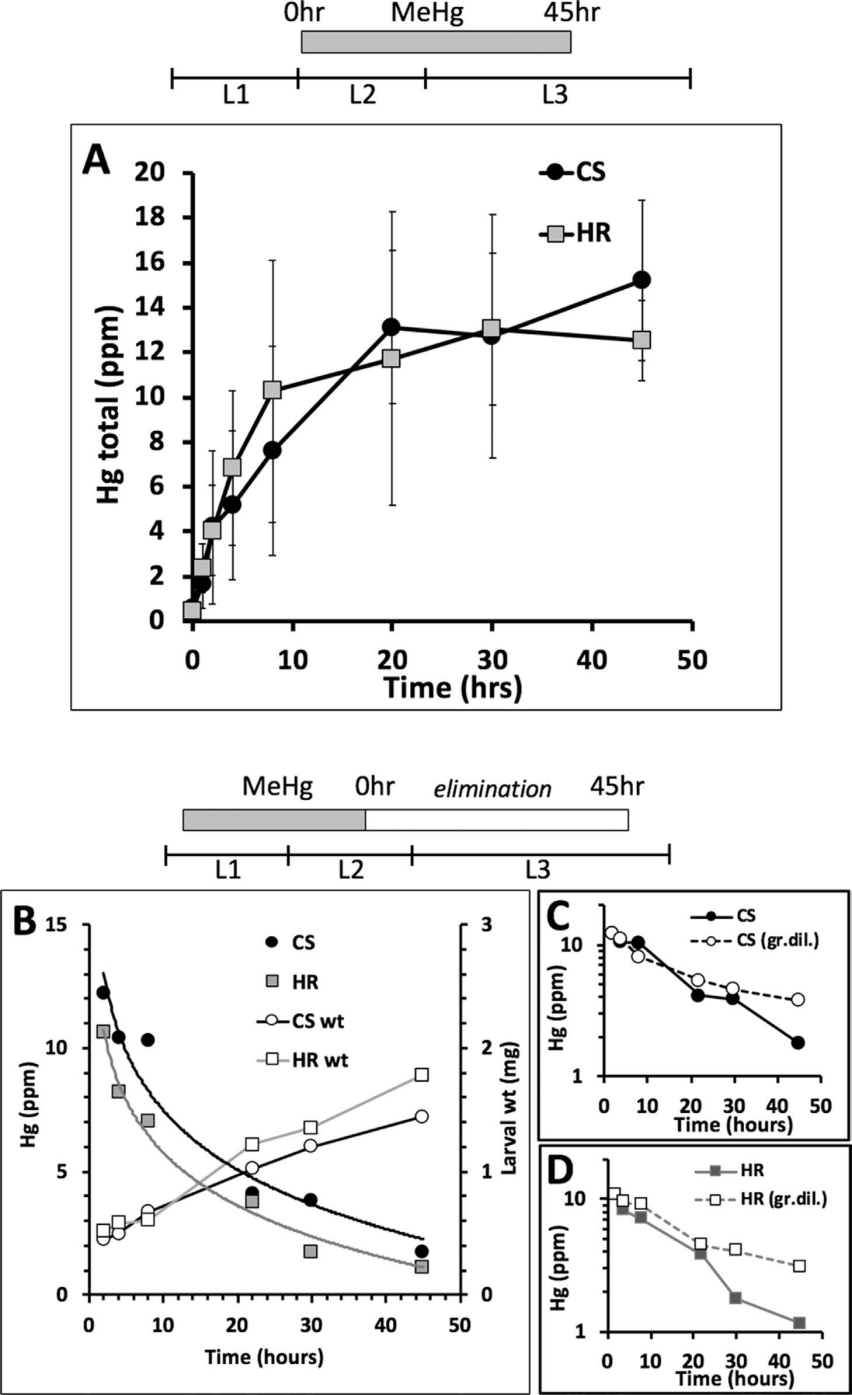
Rate of MeHg uptake and elimination. **(A)** Uptake of MeHg (5 μM in food) was initiated with early L2 larvae of Canton S (CS) and Hikone R (HR) strains and carried out for 45 h. Larvae were sampled at various time points for total Hg analysis (n = 3 replicates of pooled samples of 15 larvae/time point). **(B–D)** Elimination of MeHg was determined in larvae previously exposed to MeHg for 40 h starting at the early L1 stage. MeHg elimination was initiated by transferring L2 larvae to MeHg-free food. **(B)** Larvae were sampled at various time points (10 L3 larvae/time point) for total Hg analysis. Results from one of two trials is shown (see trial 2 in **Figure S2**). Larval weights (wt) are also plotted for each time point. **(C**, **D)** Semi-log plots of Hg versus time for CS (C) and HR (D) including predicted profile of Hg concentration over time based on growth dilution alone (dashed line, gr.dil).

We inferred from the above findings that differences in MeHg tolerance between Canton S and Hikone R could stem from body burden differences due to absorption or elimination mechanisms at the larval stage. In support of this, we saw that Canton S pupae consistently had higher levels of Hg than Hikone R pupae when reared on the same MeHg food concentrations (see **Figure 3D**). To examine a role for absorption rates in MeHg body burden we used co-administration of L-methionine, which acts as competitive inhibitor in a conserved mechanism of MeHg-cysteine conjugate uptake *via* large amino acid transporters (e.g., LAT-1, (Aschner and Clarkson, 1989; Caito et al., 2013), **Figure 3A**). Addition of L-methionine (1–5 mM) to the food medium was seen to cause a significant increase in eclosion rate of both the Canton S and Hikone R strains across several concentrations of MeHg exposure (**Figure 3B, C**; data not shown). In parallel, pupae consistently showed reduced levels of Hg body burden with L-methionine co-exposure (**Figure 3D**), consistent with an inhibition of MeHg uptake. We next compared rates of MeHg uptake in Canton S and Hikone R larvae across the L2–3 stage. The initial rate of MeHg uptake in the Canton S and Hikone R showed to be equivalent (**Figure 4A**). This is despite a large variability that was observed among the three uptake trials. This variability likely stems from the rapid growth that occurs over this developmental window (larva grow >20-fold in size over L1 to L3 stages), for which it is difficult to synchronize between trials. Nonetheless, it was apparent that a steady-state level of MeHg is reached in approximately 30–40 h in both strains at the larval stage. This is consistent with the notion that MeHg exposure initiated at the L2–early L3 stages could accomplish steady-state levels prior to pupariation.

We next examined rates of MeHg elimination from larvae fed at the L1–L2 stage and transferred to MeHg-free food. An exponential decay in Hg body burden was observed for both strains, which at first approximation, appeared nearly the same for Canton S and Hikone R (**Figure 4B**). However, a three- to fourfold increase in body weight was also seen for both strains over the elimination period (**Figure 4B**), making it necessary to account for growth dilution. For both strains, the Hg levels were seen to be reduced by half within approximately 20 h (**Figures 4C, D**). This decrease could be attributed almost entirely to growth dilution (**Figures 4C, D**, dashed lines). At the 30- to 45-h time points the Hikone R strain demonstrated a substantial drop in Hg levels indicative of elimination occurring in excess of the growth dilution rate (**Figure 4D**). In contrast, in Canton S larvae, an increase in elimination rate over the growth dilution was not seen until 45 h (**Figure 4C**). This pattern was seen across two independent trials (see **Figures S1A–C**). These higher rates of elimination in the Hikone R versus Canton S are consistent with the observation that Hikone R exhibits a lower steady-state body burden of MeHg compared to Canton S when they reach the pupal stage.

### Biotransformation of MeHg

MeHg biotransformation (demethylation) has been implicated as a rate-limiting step in the process of MeHg elimination (Rowland et al., 1978). To examine if MeHg biotransformation might be a contributing factor in MeHg elimination and tolerance, we evaluated the appearance of inorganic Hg (iHg) [relative to the total Hg (tHg)] in MeHg exposed animals during both the larval and pupal stages. First, we examined MeHg biotransformation in a “saturated” culture of Canton S larvae (40 larvae cultured in 500 µl of food for 4 days; see Methods). Second, we determined the level of biotransformation over the course of metamorphosis in both Canton S and Hikone R pupae by determining iHg as a fraction of tHg at the late pupal stage in animals reared on MeHg throughout the larval stages. In all conditions, no significant increase in iHg was observed (**Figure S2A, B**), indicating that MeHg demethylation does not occur over the course of the larval to adult development stages and is not contributing to the kinetics of elimination.

### Genetic Rescue of Eclosion *via* GCLc Elevation of GSH Levels

**Figure 5:**
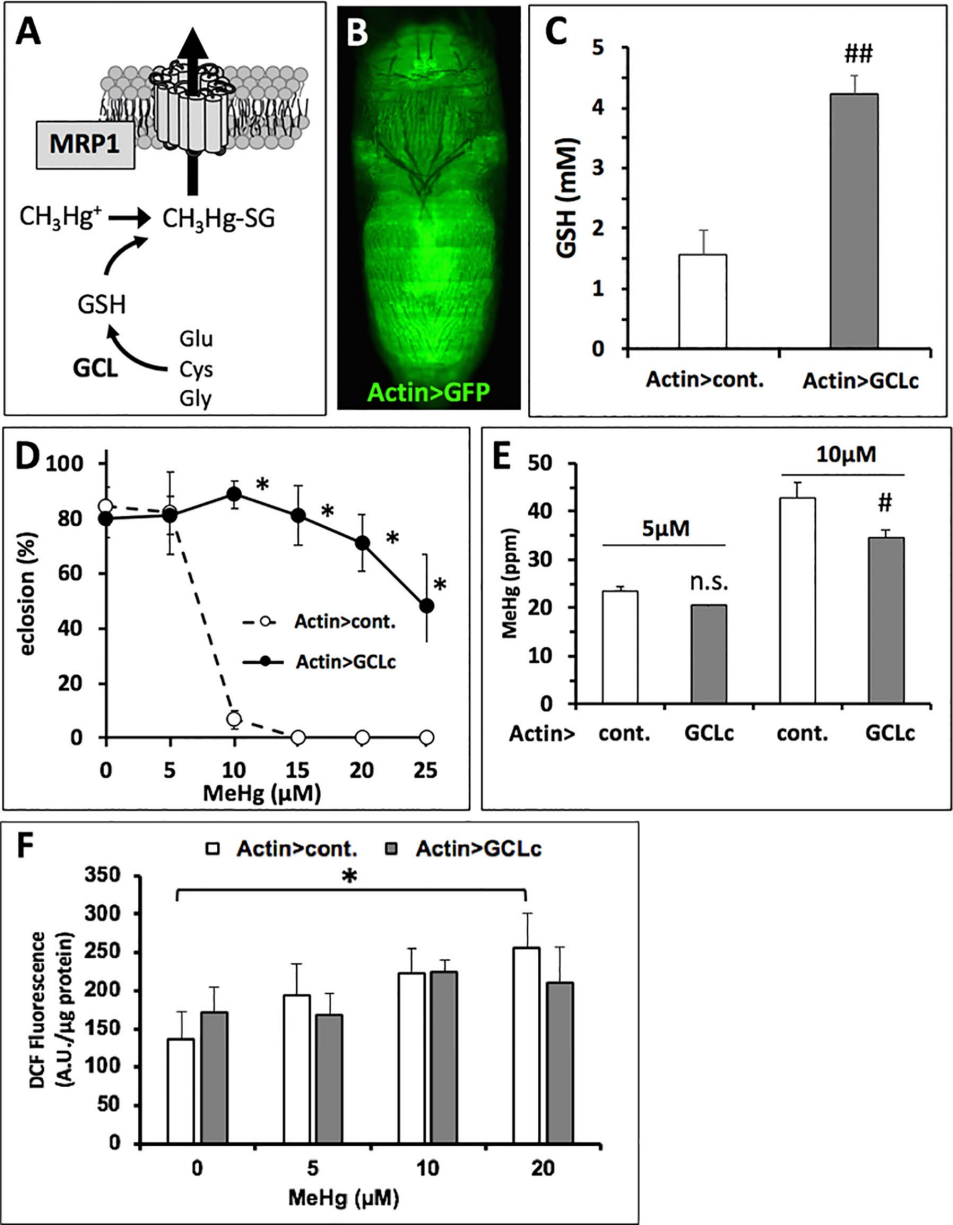
Genetic rescue of MeHg-inhibited eclosion with GCLc expression. **(A)** Glutamyl-cysteinyl ligase (GCL) is the rate-limiting enzyme in glutathione (GSH) synthesis. GSH conjugated to MeHg is a substrate for cellular export *via* the multi-drug resistance like protein transporter (MRP1) (Madejczyk et al., 2007). **(B)** Gal4 > UAS expression pattern of the actin promoter (ActinGAL4, Actin>) revealed with UASGFP in a late pupa. **(C)** Pupal GSH levels with expression of the catalytic subunit of GCL (GCLc) by the actin promoter (Actin>) compared to control (cont., the w1118 background genetic strain). GSH levels were determined in lysate preparations of whole pupae (n = 3 replicates of pooled samples of 10 pupae, ^##^ *p* < 0.0001, t-test). **(D, E)** Actin > GCLc and control (Actin > w1118) flies reared on indicated concentration of MeHg were evaluated for eclosion rate **(D**, *p < 0.0001, z-test), Hg body burden **(E**, ^#^ *p* = 0.016, t-test), and reactive oxygen species (ROS) **(F**, expressed in arbitrary fluorescence units (A.U.) per microgram protein, two-way ANOVA non-significant; one-way ANOVA, Actin > cont. *p* < 0.05 at 20 µM MeHg only, Actin > GCLc p∼nonsignificant).

The above findings indicate toxicokinetics, specifically elimination transport mechanisms, as a central determinant of MeHg tolerance and susceptibility. We next investigated the potential for genetic modulation of GSH levels to influence elimination, presumably by formation of MeHg-GSH conjugates, which are a substrate for cellular export *via* the ABC family of ATP-dependent xenobiotic transporters ((Madejczyk et al., 2007), **Figure 5A**). Using the ActinGAL4 driver for ubiquitous expression (**Figure 5B**) we achieved a nearly threefold increase in GSH levels in pupae with over-expression of the glutamyl-cysteine ligase catalytic subunit (GCLc) (**Figure 5C**). GCLc over-expression also induced a robust increase in eclosion rates of flies reared on MeHg food up to 25-µM concentration (**Figure 5D**). Hg body burden showed a lower level of Hg in pupae expressing GCLc relative to control pupae with significantly lower levels seen on 10 µM food (**Figure 5E**).

Since the robust rescue of eclosion with GCLc overexpression corresponded to only a moderate reduction in Hg body burden, we examined a potential role for GSH to act in buffering oxidative stress associated with MeHg. Using DCF-DA as a general indicator of oxidative stress resulting from ROS, we observed a dose-dependent increase in fluorescence intensity with MeHg exposure in control pupae, significant at the 20-µM MeHg treatment (Actin > cont., **Figure 5F**). In contrast, a dose-dependent increase in ROS in the Actin > GCLc pupae exposed to MeHg was not observed. Furthermore, relative to the control, the Actin > GCLc pupae did not consistently show lower levels of DCF-DA fluorescence. A two-way analysis of variance (ANOVA) showed no significant dose × genotype interaction (**Figure 5F**).

### Natural Variation in MeHg Tolerance Associated With Hg Body Burden Occurs Independent of GSH Levels

**Figure 6:**
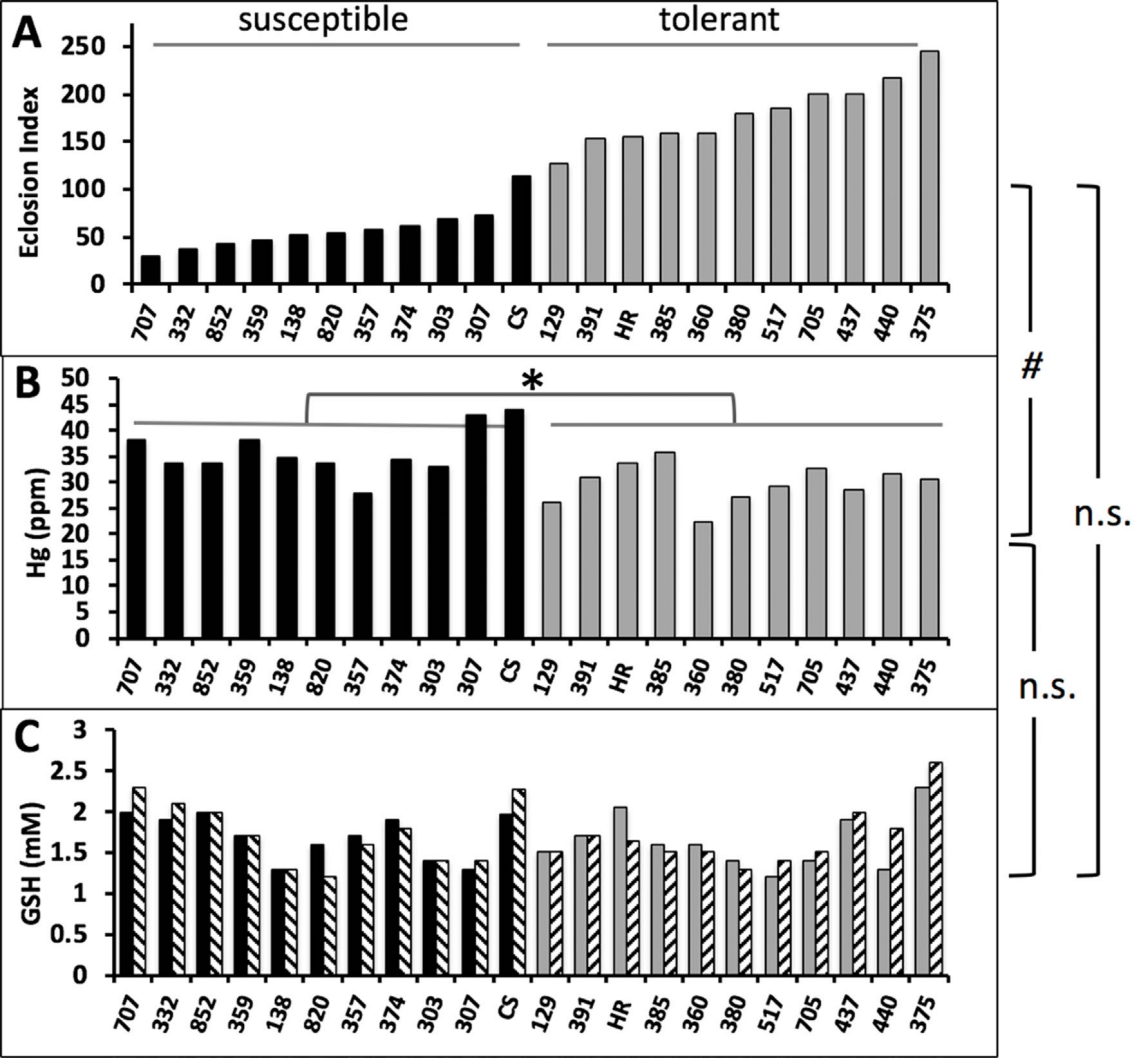
Correlations of eclosion rate, Hg body burden, and GSH levels in wild-derived strains of flies. **(A)** Eclosion rate of 10 susceptible and 10 tolerant strains of isogenic wild derived strains of flies from the DGRP panel are shown together with CS and HR. Eclosion rates are expressed as an eclosion index (see Methods) and reproduced from results in Montgomery et al. (2014). **(B)** Hg body burden was determined on tolerant and susceptible pupae reared on 10 µM MeHg food (n = 1 replicate of pooled samples of 10 pupae). Susceptible (median Hg = 34.5 ppm) and tolerant (median Hg = 30.7 ppm) groups were statistically different (*p = 0.0017, Mann-Whitney, non-parametric test). **(C)** GSH was determined in lysates of pupae (n = 1 replicate of pooled samples of 10 pupae) reared on food without (solid bars) or with (hatched bars) 10 µM MeHg. Correlation statistics were done for the relationships of eclosion index with Hg body burden [^#^ *p* = 0.043, linear regression slope = −0.0328(0.0151), C.I. −0.0643 to −0.00121], eclosion index with GSH levels (linear regression, n.s., non-significant), Hg body burden with GSH levels (linear regression, n.s., non-significant).

The above genetic manipulation of GCLc indicated that variant levels of GSH could explain individual variation in MeHg tolerance in wild populations. We tested for this trait among a subset of the most tolerant and susceptible wild-derived isogenic strains within the Drosophila Genetics Reference Panel (DGRP) collection that we have previously characterized for tolerance to MeHg (Montgomery et al., 2014). Eclosion ability, expressed as an eclosion index (see Methods), for the 10 most tolerant and 10 most susceptible strains, together with the Canton S and Hikone R strains is seen in **Figure 6A**. This was compared to Hg body burden in pupae reared on 10 µM MeHg food (**Figure 6B**) and GSH levels in pupae both with and without MeHg exposure (**Figure 6C**). Hg body burden values varied widely ranging from 22 to 43 ppm (**Figure 6B**). A significant negative correlation of Hg body burden and eclosion rate was observed [slope = −0.033 (CI, −0.064 to −0.001), *p* = 0.043]. GSH levels in pupae were also seen to vary widely between 1.2 and 2.6 mM across the 22 strains. Remarkably, across all individuals, there was no significant change in GSH levels resulting from exposure to 10 µM MeHg. Also, no significant correlation was observed between GSH levels and eclosion rate, as well as between Hg body burden and GSH levels (**Figures 6A, C**).

### Rescue of Eclosion With GCLc Expression Independent of Change in Hg Body Burden

**Figure 7:**
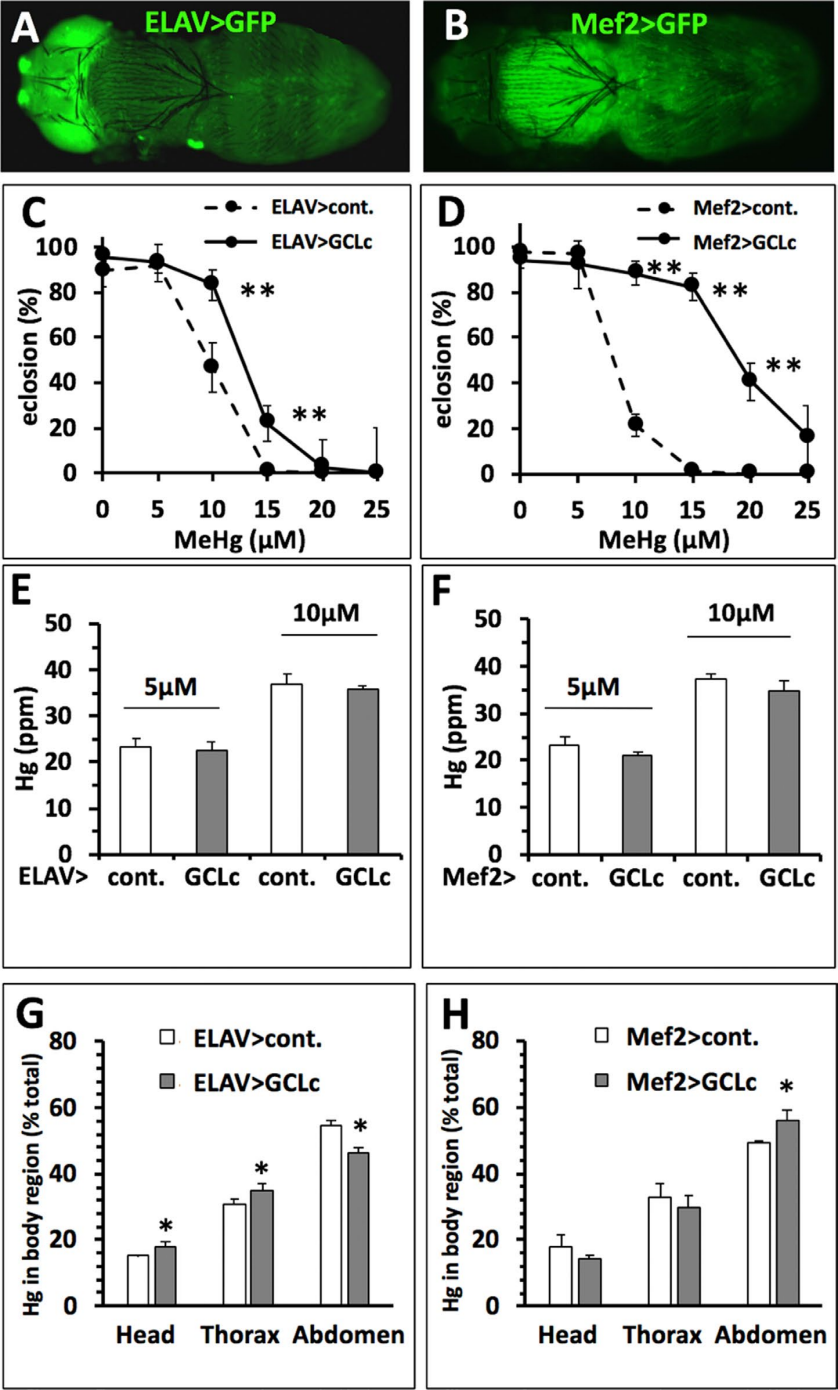
Rescue of MeHg-inhibited eclosion with no change in Hg body burden. **(A, B)** Gal4 > UAS expression pattern of drivers specific to nervous system (**A**, ELAV>) and muscles (**B**, Mef2>) in late pupal stages demonstrated in combination with UASGFP (Orientation: dorsal view, anterior to the left). **(C, D)** Eclosion with GCLc over-expression restricted to the nervous system (ELAV>), muscles (Mef2>) (*p < 0.05, **p < 0.001, z-test). **(E, F)** Hg body burden in pupae with GCLc over-expression restricted to the nervous system (ELAV>), muscles (Mef2>) (n = 3 replicates of pooled samples of 10 pupae). **(G, H)** Distribution of Hg (% of total body burden) in head, thorax, and abdomen with GCLc over-expression restricted to the nervous system (ELAV>, G), muscles (Mef2>, H) (n = 3 replicates of pooled samples of each body region dissected from 10 pupae, *p < 0.05, Wilcoxon-rank test).

The above findings suggest that, across a population, reduced Hg body burden correlates with higher eclosion rates. Nonetheless, comparisons of individual strains showed clear examples where higher Hg body burden is seen in the tolerant versus the susceptible strain [e.g., compare line #357 (E.I. = 58, Hg = 27.9 ppm) and #385 (E.I. = 159, Hg = 35.8 ppm), **Figures 6A, B**]. This condition suggests that MeHg tolerance mechanisms aside from toxicokinetic influence on body burden may exist. One possibility is that protection of the whole animal, e.g., rescue of eclosion, may rely on tolerance at the level of a specific target organ or tissue. To test this, we implemented targeted expression of GCLc under promoters specific for neurons (ELAV) and muscles (Mef2) using the Gal4-UAS system. The body region-restricted expression of these promoters can be seen at the pharate adult stage using the UASGFP reporter (**Figures 7A, B**). GCLc overexpression under both of these promoters produced a significant increase in eclosion rate on MeHg food relative to control fed pupae (**Figure 7C, D**) with GCLc expression under the muscle-specific promoter showing the greatest rescue. In contrast with ubiquitous actin-driven expression, over-expression of GCLc restricted to neurons or muscles during development showed no significant decrease in Hg body burden in pupae reared on 5 and 10 µM MeHg food compared to control pupae (**Figure 7E, F**). Since muscle and neurons are known targets of MeHg toxicity, we examined whether this increased tolerance with targeted expression GCLc was due to a redistribution of MeHg away from these tissues. Hg levels were determined in the head (neuron enriched), thorax (muscle enriched), and abdomen of pupae reared on 10 µM MeHg food. GCLc over-expression under the ELAV neural promoter showed a significant shift in Hg levels, unexpectedly, with increased Hg seen in the head and thorax and a corresponding decrease in the abdomen (**Figure 7G**). In contrast, muscle targeted (Mef2) GCLc expression showed a trend toward reduced levels of Hg in the head and thorax and a significant increase in Hg in the abdomen (**Figure 7H**).

## Discussion

We have used a Drosophotoxicology paradigm to elaborate traits that highlight distinct toxicokinetic and toxicodynamic modes of action of MeHg. First, we resolve that the pupal stage is the most sensitive to MeHg toxicity with respect to other developmental windows in the fly life cycle. Through a series of dosing paradigms and kinetic analyses, we resolved that MeHg tolerance or susceptibility is inversely related to the Hg body burden in the pupa, which, in the case of two well-characterized strains, Cantons S and Hikone R, can be attributed to differences in elimination rates at the larval feeding stage. Consistent with a mechanism where MeHg transport and elimination is facilitated by GSH, we demonstrate a robust rescue of MeHg toxicity with increased GSH levels that also correlates with a decrease in Hg body burden. However, this relationship of increased tolerance with increased GSH levels and decreased Hg body burden is not consistent among all individuals of a select population of wild-derived flies from the DGRP. Instances are seen where higher Hg body burden in tolerant flies compared to susceptible flies. Furthermore, we demonstrate, with tissue restricted expression of GCLc that robust tolerance can be achieved with no significant decrease in Hg body burden, clearly indicating a toxicodynamic mechanism capable of constituting MeHg tolerance.

The difference in MeHg tolerance between the Canton S and Hikone R strains can be explained by toxicokinetics. L-methionine co-exposures in both these strains illustrate the direct relationship of lower Hg body burden and increased eclosion rates. Inhibition of uptake by L-methionine also indicates conserved mechanisms of MeHg uptake by L-type amino acid transporters (Aschner and Clarkson, 1989; Caito et al., 2013) exist in the fly and that variability in toxicity could arise from genetic variation, for example, in the LAT1 transporter. However, rates of MeHg uptake were the same for both the Canton S and Hikone R strains, and the lower steady-state Hg levels in Hikone R were seen to relate to a faster rate of MeHg elimination in Hikone R in late larval life. This finding predicts that a MeHg tolerance trait may be attributed to genetic variants of xenobiotic transporters that mediate MeHg excretion. Consistent with this, over-expression of MRP1 (ABCC1), a known transporter for MeHg, in flies gives robust MeHg tolerance (Prince and Rand, 2017). Alternatively, flies carrying a mutant allele for MRP1 are more susceptible to MeHg and show higher levels of MeHg accumulation (Prince et al., 2014). Nonetheless, compared to the Canton S strain, the Hikone R strain shows slightly lower basal expression of MRP1 (Prince et al., 2014). Thus, other mediators of MeHg transport are likely involved in the kinetics of MeHg toxicity.

We find that MeHg biotransformation (demethylation) does not occur in Drosophila. MeHg demethylation is an integral part of the kinetics of MeHg elimination in mammals and is likely a determinant of individual variation in Hg body burden (Rand and Caito, 2019). Thus, the absence of MeHg biotransformation in flies could be seen as a limitation of this study, where it is possible that kinetic mechanisms involving biotransformation in mammals may have a greater overall influence on MeHg tolerance and toxicity than toxicodynamic mechanisms. Alternatively, the absence of MeHg demethylation activity in Drosophila could be viewed as an opportunity to exploit this model once again to characterize such activity in the future *via* introducing it to transgenic flies.

Our results also elucidate that toxicodynamic pathways can influence MeHg toxicity independent of kinetic influences on Hg body burden. Ubiquitous (Actin>) expression of GCLc significantly raises systemic GSH levels and can alter kinetics as seen by a lowering of Hg body burden and a corresponding greater eclosion rate compared to control pupae. However, when contrasted with the relationship of eclosion rate and Hg body burden seen in the Canton S and Hikone R strains, this lowering of Hg body burden with GCLc overexpression is far too modest to explain the robust rescue of eclosion that is seen. A dose-dependent rise in oxidative stress with MeHg is seen with DCF-DA fluorescence in lysates of control pupae, but is not seen with GCLc expressing pupae, suggesting that an increase in GSH can indeed buffer ROS resulting from MeHg. Nonetheless, the magnitude of ROS induction with MeHg, and the lack of significant dose X genotype effect, indicates that ROS production, while a contributor, does not comprise a major mediator of MeHg toxicity in this system. Curiously, when GCLc over-expression is restricted to specific tissues Hg body burden does not decrease yet eclosion substantially increases. This may reflect a mechanism whereby tissue restricted elevation of ROS occurs that is effectively buffered with GCLc/GSH targeted to that tissue. Another likely mechanism is that tissue restricted GCLc/GSH expression preferentially redistributes MeHg away from sensitive targets. Consistent with this, GCLc expressed in muscle, which is most abundant in the thorax due to the indirect flight muscles, causes a trend toward reduced Hg levels in the thorax and head with a significant increase of Hg in the abdomen. Yet, unexpectedly, we see the opposite effect when GCLc over-expression is targeted to the nervous system, which causes a slight, but significant, redistribution of Hg to the head and thorax with a corresponding reduction of Hg in the abdomen. A possible explanation for this latter observation could be that an increased level of GSH in brain tissue and the thoracic ventral nerve cord sequesters MeHg to the head and thoracic regions in the form of MeHg-SG conjugates. At the same time, these MeHg-SG complexes may act subcellularly to sequester MeHg away from sensitive intracellular thiol targets in neural tissues. The overall elevation of Hg in neural tissues that results may be due to limiting rates of transport of MeHg-SG out of neural cells. By comparison, muscle cells may have a greater intrinsic activity to export MeHg-SG, causing redistribution from the thorax to the abdomen. Together, these genetic manipulations of GCLc highlight underlying complexity of toxicodynamic mechanisms that can potentially act in a selective manner in distinct tissue targets to give MeHg tolerance.

We also see evidence for contrasting traits of kinetics and dynamics influencing MeHg tolerance in wild populations. A grouped analysis of the 10 most MeHg-tolerant and 10 most MeHg-susceptible wild-derived isogenic lines within the DGRP panel showed that the tolerant strains, on average, exhibited lower levels of MeHg body burden compared to the susceptible strains. While this would argue that tolerance is dictated by kinetics (e.g., faster elimination rates leading to lower body burden), this trait did not correspond with systemic GSH levels. Furthermore, several individual strains did not adhere to this relationship; for example, DGRP tolerant strain #385 gives a much higher eclosion index than susceptible strain #357, yet #385 exhibits higher Hg levels and slightly lower GSH levels than #357. This profile suggests that it is toxicodynamic handling of MeHg, independent of GSH, in strain #385 that preserves eclosion behavior and supports its higher MeHg tolerance.

## Conclusions

These data support a model whereby MeHg tolerance can arise from either toxicokinetic determinants, i.e., enhanced excretion and elimination of MeHg from the body, or alternatively, toxicodynamic determinants, i.e., selective sequestration of MeHg or buffering of ROS at sensitive sites of toxic action in target organs, such as muscle and neurons. This finding has important implications for future studies of MeHg toxic potential in populations, and potentially for clinical applications, where the objective is to relate a measurement of Hg body burden (e.g., Hg measured in hair or blood) to a developmental outcome. It is possible that two individuals with the same body burden of Hg could be genetically predisposed to starkly different toxicity outcomes based on genetic determinants of a toxicodynamic process. Future studies utilizing the experimental model detailed here, where toxicodynamics and toxicokinetic determinants can be clearly discerned, are required to elucidate the key factors mediating MeHg toxicodynamics and inform methods to identify genetic signatures of MeHg tolerance and susceptibility.

## Author Contributions

MR, DV, LP, AP and JG conceived and executed the experiments. MR and LP wrote the manuscript.

## Funding

R01ES025721 (MR—P.I.); Rochester Environmental Health Science Center P30ES001247; R01ES010219 (MR Co-I.); T32ES007026 (LP, JG, AP).

## Conflict of Interest Statement

The authors declare that the research was conducted in the absence of any commercial or financial relationships that could be construed as a potential conflict of interest.
