# Supplementary figures and images for: Drosophotoxicology: Elucidating Kinetic and Dynamic Pathways of Methylmercury Toxicity in a Drosophila Model
Source: Front Genet. 2019 Aug 9;10:666. doi: 10.3389/fgene.2019.00666 (PMC6695472; doi:10.3389/fgene.2019.00666)

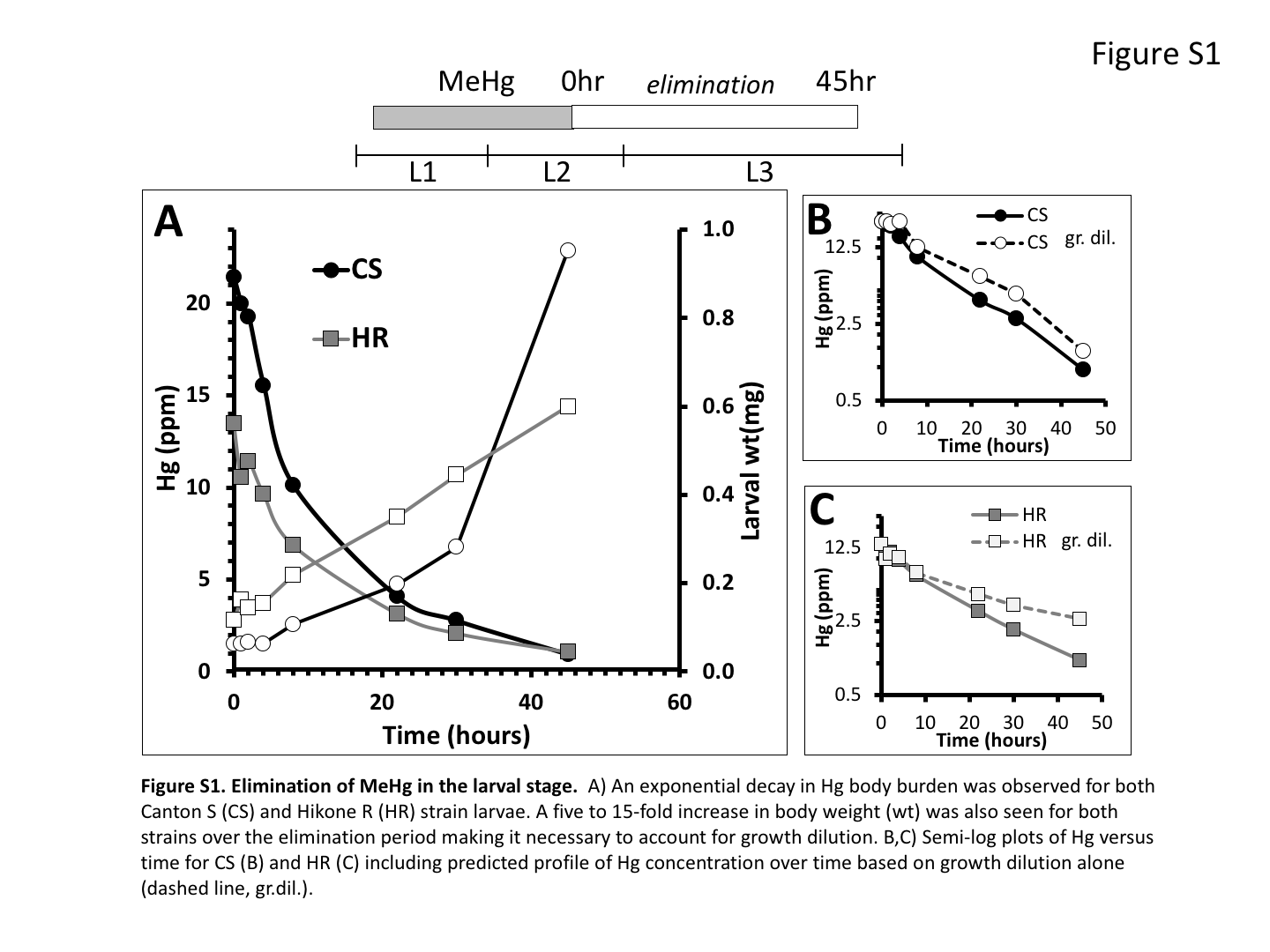

Supplement: Supplementary file 1 [file Image_1.tiff]

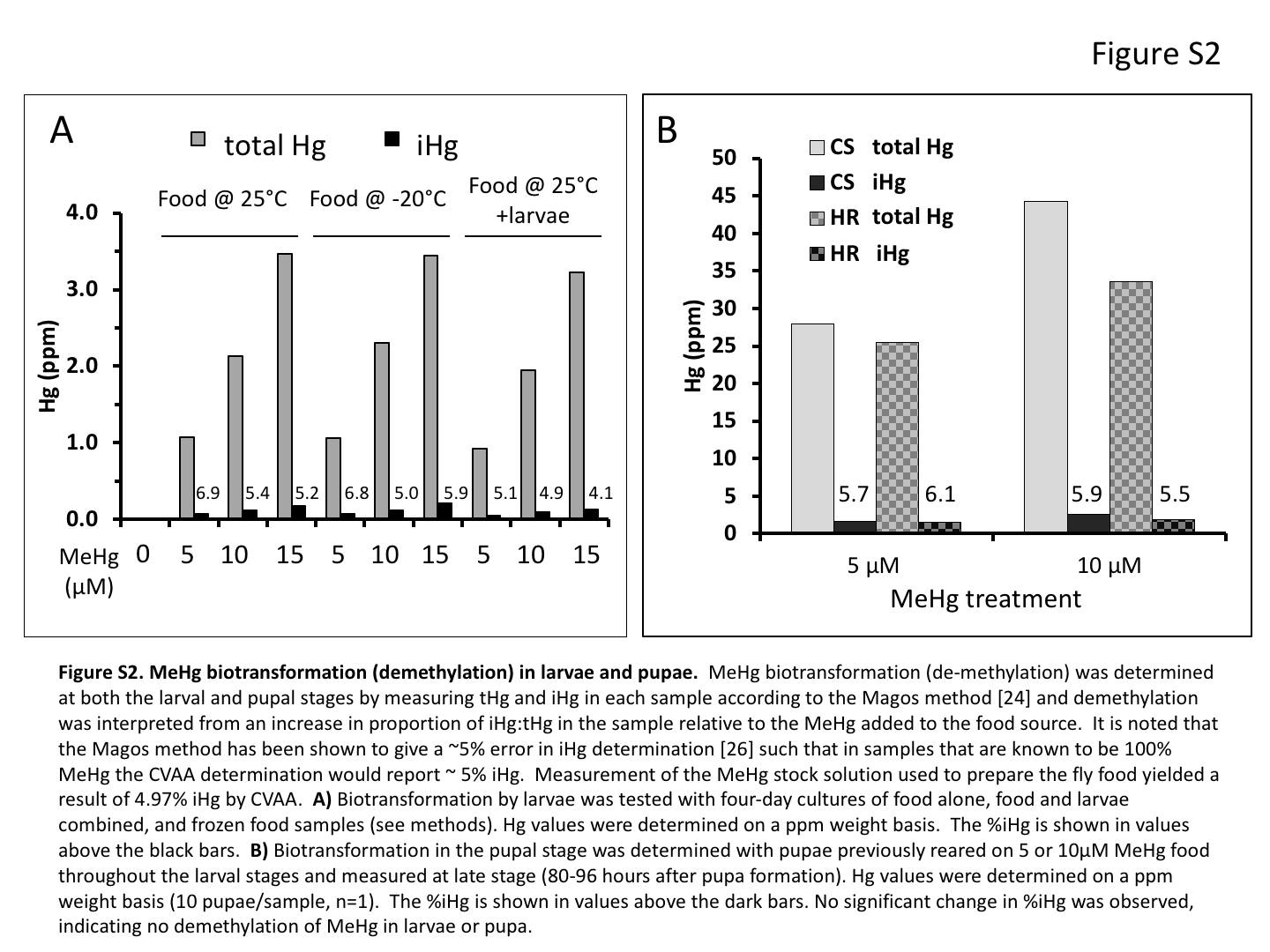

Supplement: Supplementary file 2 [file Image_2.tiff]
